# Bellevue Hospital

**Published:** 1857-10

**Authors:** 


*Bellevue Hospital.*—The extensive enlargement of the buildings occupied by this charity, are in rapid progress. For the present we learn that the lack of room for the inmates is sadly felt. Indeed, all our institutions are unusually full for the season. The Lunatic Asylum, on Blackwell's Island, is so filled, that an additional building has been occupied. The Alms House buildings, too, are crowded.—*The American Medical Gazette.*

